# Mechanisms of pain occurrence in osteoarthritis: peripheral triggers, sensitization, and the path to persistence

**DOI:** 10.3389/fimmu.2026.1820875

**Published:** 2026-06-26

**Authors:** Kai Huang, Haili Cai

**Affiliations:** 1 Department of Orthopaedics, Tongde Hospital of Zhejiang Province, Hangzhou, China; 2 Department of Ultrasound, The 903rd Hospital of the People’s Liberation Army, Hangzhou, China

**Keywords:** neurovascular remodeling, osteoarthritis pain, pain occurrence, peripheral sensitization, synovial immune dysregulation

## Abstract

Osteoarthritis (OA) is a joint disease, and pain drives disability. Radiographic severity correlates poorly with pain intensity, indicating that OA pain cannot be explained by cartilage loss alone. This narrative review focuses on OA pain occurrence, the events that translate joint pathology into episodic activation of nociceptive pathways. We synthesize evidence that pain occurrence is initiated and shaped by peripheral mechanisms, including algogenic signaling in tissues, access of nociceptors to pain sources, and sensitization related lowering of activation thresholds. Key triggers include inflammatory and lipid mediators, neurotrophin-dependent sensitization, and joint acidosis. Proton-sensing channels and receptors are implicated in pH-driven nociceptor activation (preclinical evidence), though direct causal demonstration in human OA remains limited. Neurovascular remodeling and sympathetic-sensory crosstalk have been associated with increased nerve density and excitability in preclinical models, and synovial immune dysregulation may contribute to a permissive microenvironment, with human evidence largely observational or inferential. Systemic factors, particularly obesity and metabolic dysfunction, bias joint biology toward heightened pain responsiveness via adipokines and inflammation. Persistent peripheral input may promote central sensitization and pain chronicity. We discuss implications of targeting peripheral mechanisms to reduce pain occurrence and prevent transition to refractory, centrally amplified pain states. Inferences are limited by cross-sectional designs, intensity-dominant outcomes, limited phenotype stratification, and translational gaps between experimental models and the prolonged course of OA in humans. Peripheral and central processes are often inferred indirectly and co- occur, complicating attribution along pain trajectories. Longitudinal, stage and phenotype stratified cohorts integrating imaging, molecular and sensory phenotyping, plus occurrence oriented outcomes, are needed.

## 1 Introduction

Osteoarthritis (OA) is the most prevalent chronic joint disease worldwide and a leading cause of pain and disability in aging populations (1). Although OA is characterized by articular cartilage degeneration, subchondral bone remodeling, and synovial alterations, pain is the main driver of patient suffering, functional impairment, and health-care utilization (2). Notably, clinical trials and cohort studies have shown that radiographic and histopathological severity often correlates weakly with pain intensity, emphasizing that pain cannot be solely attributed to tissue degeneration (3–5), indicating that OA pain cannot be attributed to tissue degeneration alone. We note, however, that this structure-pain discordance is not explained only by joint-local biology. It may also reflect the limited sensitivity of conventional radiography for pain-relevant pathology, as well as modulation by psychosocial factors, comorbid widespread pain, sleep disturbance, mood, and treatment exposure. Although these broader contributors are important, the present review focuses on the peripheral and joint-centered biological mechanisms underlying pain occurrence. “Pain occurrence” refers to a set of biological events that translate joint pathology into the initial and recurrent activation of pain pathways. It focuses on the early biological events that initiate pain, while nociception refers to the sensory detection of harmful stimuli, and pain experience refers to the subjective perception of pain. Specifically, pain occurrence includes: (i) the generation of algogenic signals within the joint microenvironment, (ii) the access of sensory nerves to pain-generating regions, and (iii) the lowering of nociceptor activation thresholds through sensitization processes.

OA pain is now understood as a continuum, evolving from episodic, activity-related pain to persistent, chronic pain, which may involve neuropathic-like or nociplastic features as the disease progresses. This evolution is marked by complex mechanisms, including peripheral nociceptor activation, neuronal sensitization, neurovascular remodeling, immune dysregulation, and central nervous system plasticity (6). Traditionally, OA pain was considered a predominantly nociceptive phenomenon driven by mechanical overload and inflammation of innervated joint tissues such as the synovium, periosteum, ligaments, and subchondral bone. However, accumulating evidence indicates that OA pain is biologically heterogeneous and mechanistically dynamic, involving a complex interplay between peripheral nociceptor activation, peripheral neuronal sensitization, neurovascular remodeling, immune dysregulation, and-at later stages-central nervous system plasticity. Consequently, OA pain is now understood as a continuum, evolving from episodic, activity-related pain to persistent, treatment-resistant pain with neuropathic-like or nociplastic features.

**Figure 1:**
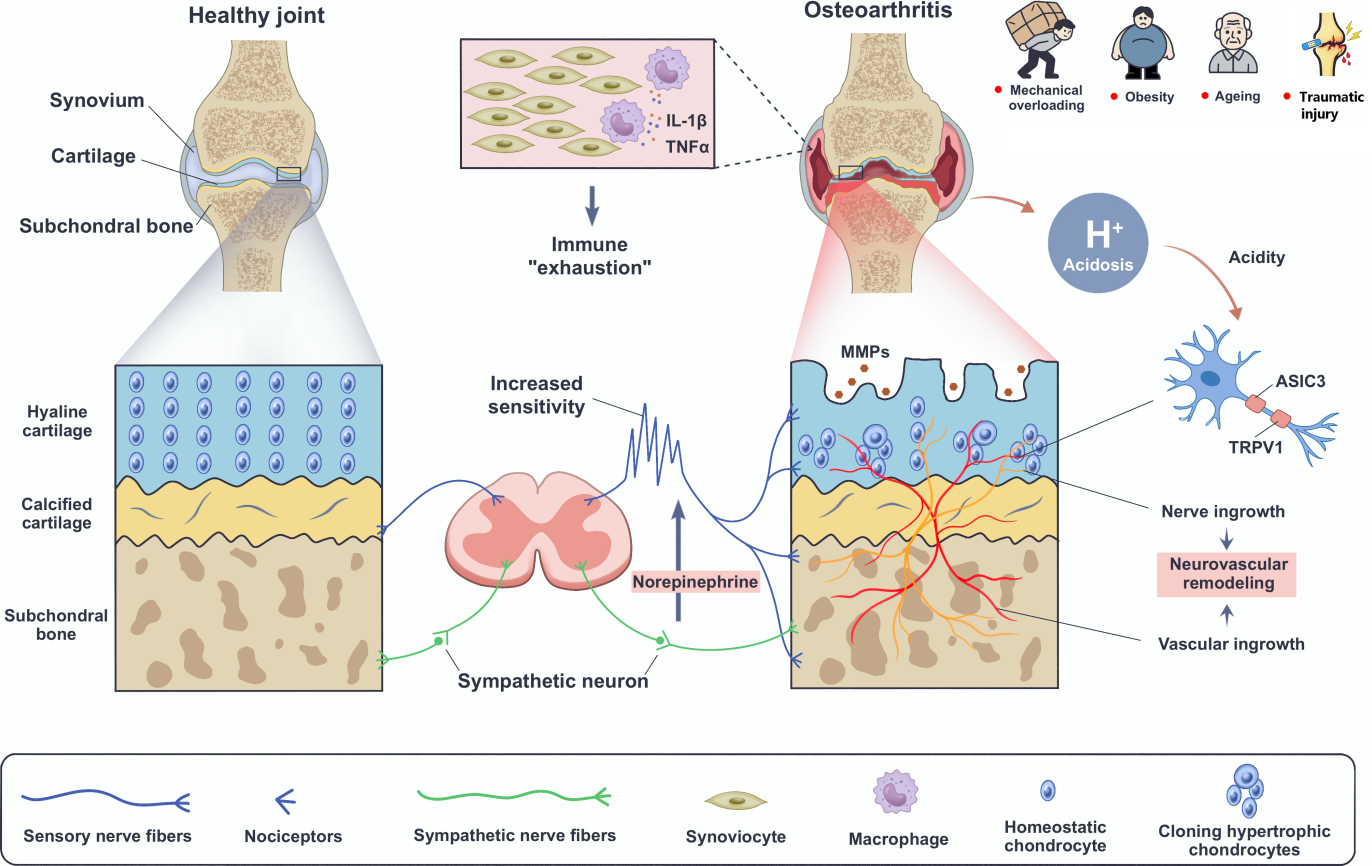
Mechanistic framework of pain occurrence and persistence in osteoarthritis. Joint pathology in innervated tissues generates algogenic mediators and local acidosis that activate proton-sensitive nociceptors, initiating pain occurrence. Repeated stimulation induces peripheral sensitization, lowering activation thresholds and increasing pain frequency. In parallel, metabolic, immune, and chemical drivers-including obesity-related metabolic dysfunction, synovial immune dysregulation, and inflammatory or lipid mediators-amplify nociceptor excitability and sustain a pain-permissive microenvironment. Neurovascular remodeling and sympathetic-sensory crosstalk further enhance neural access and responsiveness. Through these interacting mechanisms, sustained peripheral input promotes central sensitization, driving the transition from episodic pain occurrence to persistent and chronic osteoarthritis pain.

Recent studies have implicated several possible, previously underappreciated contributors to OA pain occurrence. These include joint acidosis (7) and proton-sensing receptors (8), neurotrophin-dependent peripheral sensitization (9), neurovascular remodeling (6) and possible sympathetic–sensory interactions (10), and synovial immune dysregulation. Rather than representing established causal drivers in isolation, these processes may shape a pain-permissive neuro-immune-vascular microenvironment that modulates nociceptive signaling. In parallel, systemic factors such as obesity and adipose tissue dysfunction modulate local joint biology, biasing the nociceptive system toward heightened pain responsiveness (11). Together, these mechanisms challenge the traditional view of OA pain as a passive consequence of tissue damage, positioning it instead as an actively regulated neuro-immune-vascular process, where interactions between peripheral, immune, and vascular factors modulate nociceptive signaling (**Figure 1**).

The aim of this review is to synthesize current evidence on the mechanisms underlying the occurrence of pain in OA, with a primary focus on peripheral and joint-centered processes that initiate and amplify nociceptive signaling. By integrating findings from studies on neuronal sensitization, neurovascular remodeling, immune dysregulation, metabolic influences, and emerging therapeutic targets, this review seeks to provide a mechanistic framework that bridges joint pathology and clinical pain. Such an understanding is essential for the development of mechanism-based, precision interventions that not only relieve pain but also prevent its persistence in OA.

## 2 Literature search strategy

This review is a narrative synthesis informed by a structured literature search rather than a systematic review. To ensure comprehensive coverage of key mechanistic domains underlying OA pain occurrence, searches were conducted in PubMed/MEDLINE, Embase, and the Web of Science Core Collection from database inception through 31 January 2026. We combined controlled vocabulary (MeSH/Emtree) and free-text terms covering three primary concepts: (1) osteoarthritis, (2) pain phenotypes, and (3) mechanisms relevant to nociception, sensitization, inflammation, neural remodeling, and metabolism. In addition, targeted searches were performed for specific mechanistic pathways, including proton-sensing channels, NGF-TrkA signaling, neurovascular remodeling, sympathetic-sensory interactions, synovial immune dysregulation, and metabolic inflammation. Reference lists of included articles and relevant reviews were also screened for additional studies. Studies were included based on relevance to OA pain mechanisms, with a focus on factors influencing pain occurrence and peripheral-to-central sensitization pathways. Both preclinical and clinical studies were considered, with human studies prioritized in the synthesis whenever available. Only English-language studies were included. Screening and selection were performed by reviewing titles, abstracts, and full texts to identify studies that provided mechanistic insights or experimental evidence relevant to OA pain. As this is a narrative review, formal risk-of-bias or quality assessment was not conducted. The aim of this approach was to integrate mechanistic knowledge from multiple evidence streams-including observational human studies, interventional trials, animal experiments, and *in vitro* investigations-into a coherent framework linking joint pathology to pain occurrence.

## 3 OA pain phenotypes and clinical anchors for “occurrence”

OA pain is heterogeneous and reflects distinct, yet often overlapping, mechanistic-clinical states across disease stages. In this review, pain phenotype refers to a broad pattern in which a particular pain mechanism, or combination of mechanisms, appears to predominate, such as nociceptive-dominant, neuropathic-like, nociplastic, or mixed states. By contrast, clinical anchors are the observable clinical features or assessment domains that help infer which phenotype may be present in a given patient. In this sense, phenotypes represent latent mechanistic-clinical states, whereas anchors serve as practical indicators used to approximate them. This distinction is particularly relevant for understanding pain occurrence, defined here as the processes by which joint pathology is translated into episodic or recurrent activation of nociceptive pathways. Phenotyping OA pain therefore provides a framework for linking clinical presentation to the biological triggers that may underlie pain onset and fluctuation (**Table 1**).

**Table 1:** Mechanistic contributors, clinical anchors, biomarkers, and representative evidence in OA pain.

Mechanistic contributor	Suggestive clinical anchors	Candidate biomarkers	Evidence context
Peripheral sensitization	Activity-related pain that becomes more frequent; lower pain threshold; pain after ordinary movement	QST-derived sensory biomarkers; NGF/TrkA-related mediators; nociceptor ion-channel markers	Mainly preclinical and translational evidence, with clinical inference from sensitization-related pain patterns (6, 9, 31, 33, 34, 47)
Neurovascular remodeling and sympathetic-sensory crosstalk	Neuropathic-like symptoms; persistent or disproportionate pain; possible pain despite modest inflammatory findings	MRI bone marrow lesions/subchondral vascular features; vascular or neural imaging markers; angiogenesis/nerve-growth markers	Largely preclinical and mechanistic evidence; human causal evidence remains limited (6, 10, 35, 36)
Synovial immune dysregulation	Pain associated with synovitis; inflammatory flares; rest or nocturnal pain in some patients	Contrast-enhanced MRI synovitis scores; inflammatory mediators; macrophage/fibroblast activation signatures	Human observational evidence for synovitis-pain associations, supported by translational and mechanistic studies (4, 19, 38–42)
Obesity and metabolic dysfunction	Increased pain susceptibility; greater nociceptive responsiveness; pain disproportionate to structural damage	Adipokines; cytokines/systemic inflammatory markers; metabolic and adiposity-related indices	Clinical and mechanistic evidence supports obesity and adipose dysfunction as pain amplifiers (11, 43–45)
Joint acidosis and proton-sensitive signaling	Episodic pain linked to loading or local inflammatory/metabolic stress	Joint pH or acidoCEST-UTE MRI signal; ASIC/TRPV/proton-sensing GPCR markers; acid-related metabolic markers	Human imaging and mechanistic/preclinical evidence support plausibility, but direct human causal evidence remains limited (7, 24, 27–31)

References are representative rather than exhaustive. Clinical anchors are suggestive features used to support mechanistic inference and should not be interpreted as diagnostic indicators of a single pain mechanism.

Mechanistically, OA pain is often viewed as a combination of nociceptive, neuropathic-like, and nociplastic components. In many patients, especially early in the disease course, pain is predominantly nociceptive, arising from activation of peripheral nociceptors in innervated joint tissues such as the synovium, subchondral bone, and periosteum by mechanical stress and local biochemical mediators. This phenotype is typically intermittent and activity-related, consistent with animal studies suggesting that pain occurrence is a state-dependent response to changes in the joint microenvironment (12). With repeated nociceptor activation, however, activation thresholds may decrease and peripheral sensitization may develop, making pain more frequent and less tightly coupled to joint loading (13, 14).

In other patients, neuropathic-like symptoms, including burning pain, electric shock-like sensations, or paresthesia, suggest involvement of somatosensory system dysfunction (15). Others exhibit features more consistent with nociplastic pain, such as pain hypersensitivity and widespread pain, indicating altered central pain processing (16). These components may coexist with ongoing peripheral nociceptive input, resulting in mixed phenotypes and further complicating interpretation of structure-pain relationships. Several clinical anchors may help infer the dominant mechanisms underlying OA pain occurrence. However, these features should be considered suggestive rather than diagnostic of specific mechanisms. Temporal pain patterns may provide useful clinical anchors for mechanistic inference, although they should not be interpreted as diagnostic of a single mechanism. Episodic, load-dependent pain is more consistent with ongoing peripheral nociceptive triggers, whereas persistent pain, rest pain, or nocturnal pain may suggest sustained sensitization, central amplification, or active joint inflammation/flare states, depending on the broader clinical context (16, 17).

Symptom quality, including neuropathic-like descriptors, also provides useful clues. Although WOMAC and KOOS capture pain severity and functional impact, they do not resolve underlying mechanisms; incorporating neuropathic symptom questionnaires, quantitative sensory testing, and assessment of widespread tenderness may support or strengthen mechanistic inference and pain phenotyping, particularly for sensitization-related or neuropathic-like features, although these approaches are not diagnostic in isolation (15). Imaging, particularly MRI, can further anchor pain to joint-level biological processes such as synovitis, bone marrow lesions, or vascular changes, whereas marked discordance between imaging findings and pain may suggest sensitization-dominant states (18–20).

Overall, OA pain phenotypes should be viewed as dynamic and overlapping rather than fixed categories. Clinical pain patterns, symptom features, sensory testing, and imaging findings may collectively support inference about the dominant mechanisms driving pain occurrence, although the extent to which these phenotypes map onto specific biological drivers, and how they evolve over time, remains uncertain. Improved phenotype-based stratification may therefore facilitate earlier, more mechanism-informed interventions before persistent and centrally amplified pain becomes established.

## 4 Peripheral triggers of pain occurrence within the joint microenvironment

Peripheral pain occurrence in OA is driven by changes in the joint microenvironment, where pathology in innervated tissues, particularly the synovium and subchondral bone, is translated into nociceptive input. These processes involve chemical triggers, altered neuronal excitability, structural remodeling, and loss of local homeostatic control, which together determine when and why pain is initiated at the joint level. The following sections consider these mechanisms in an integrated manner, with emphasis on their distinct contributions and interactions.

### 4.1 Innervated joint tissues as primary generators of OA pain

Although OA is often framed as a cartilage disease, pain arises predominantly from innervated joint compartments rather than cartilage itself. Articular cartilage is aneural and avascular and therefore is not itself a direct source of nociceptive input. Instead, the synovium, subchondral bone, periosteum, capsule, ligaments, and menisci contain sensory and autonomic fibers capable of detecting mechanical deformation, biochemical stress, and inflammatory signals (21). As OA develops, these tissues undergo remodeling (fibrosis, marrow changes, microdamage, altered pressure dynamics) that increases nociceptive drive. Importantly, pain can occur before overt structural destruction becomes apparent (22, 23), suggesting that relatively modest alterations in pain-relevant, innervated joint tissues may be sufficient to initiate pain episodes. In OA, this is particularly plausible for the synovium and subchondral bone, which are well-recognized sources of nociceptive input. A useful framing is that OA pain “starts where nerves are”: joint pathology becomes painful when it intersects with neural terminals and their local microenvironment. This tissue-centric map also explains clinical variability: identical radiographic severity can produce different pain burdens depending on which structures are involved and how strongly they are innervated. Finally, structural access to pain-generating niches is not fixed. Disease-associated changes in tissue architecture and local signaling can permit nerves to infiltrate regions that were previously less accessible, thereby increasing the probability that mechanical load or biochemical shifts will be transduced into pain. In this view, peripheral pain occurrence reflects the convergence of (i) a vulnerable innervated tissue, (ii) a permissive local milieu, (iii) a neural system primed to respond, and (iv) a precipitating mechanical, inflammatory, or metabolic perturbation that is sufficient to trigger nociceptor activation.

### 4.2 Chemical and metabolic triggers: algogenic milieu and joint acidosis

A defining feature of the osteoarthritic joint is a chemical milieu enriched in mediators that either directly excite nociceptors or render them more responsive. Neurotrophins, chemokines, and lipid mediators act as molecular translators, converting tissue stress into neural firing. Within this broader chemical landscape, clinical studies have shown that joint acidosis represents a particularly direct trigger of pain occurrence in OA patients (24). Reduced pH can arise from inflammation (25), hypoxia (26), altered cellular metabolism, and impaired clearance, creating microdomains where sensory terminals are repeatedly exposed to proton-driven excitation.

Nociceptors express multiple proton-sensitive detectors, including ASICs (notably ASIC3) (27), TRP channels (e.g., TRPV1/TRPA1) (28, 29), and proton-sensing G-protein-coupled receptors (GPCRs) (30). Acidic shifts can depolarize nociceptors and modulate responses to other stimuli in preclinical models. While acidosis is a plausible contributor to pain occurrence in OA, direct evidence for causality in humans is limited and mostly inferential. Because acidic stress often coexists with inflammatory and lipid mediators, its effects are likely to interact with broader excitability pathways rather than act in isolation (31). This provides a mechanistic basis for recurrent pain episodes even when overt structural progression appears limited.

### 4.3 Peripheral neuronal sensitization in the transition to frequent pain

Peripheral sensitization represents the functional consequence of repeated joint-derived nociceptive stress. Although transient increases in nociceptor sensitivity may be adaptive by protecting the joint from further stress and facilitating recovery, repeated exposure to inflammatory, metabolic, or mechanical stimuli in OA can drive a more persistent state, lowering nociceptor activation thresholds through receptor and ion-channel sensitization, downstream intracellular signaling, and longer-term phenotypic changes that sustain heightened responsiveness (9, 31–34). Clinically, sensitization explains the transition from pain that occurs primarily with high load to pain that arises during ordinary movement or persists beyond activity. It also creates a feed-forward loop: increased nociceptor activity promotes release of neuropeptides and additional local mediators, which further amplify joint excitability. Importantly, peripheral sensitization is not synonymous with central sensitization; it is a joint-proximal process that can occur early and remains a key driver of pain occurrence even before widespread hypersensitivity develops (34). This makes it an attractive therapeutic target, as modulating peripheral excitability may reduce the frequency of pain episodes and slow progression toward more refractory pain states. Conceptually, sensitization is the “memory” of the joint’s noxious environment, embedding past inflammatory/metabolic stress into a nervous system that is now primed to respond too strongly and too often.

### 4.4 Neurovascular remodeling and sympathetic–sensory crosstalk

Beyond chemistry and sensitization, OA pain occurrence is enabled by structural and autonomic mechanisms that amplify and stabilize peripheral nociceptive input. Neurovascular remodeling, including coordinated angiogenesis and nerve growth, is associated with increased innervation in preclinical models, potentially supporting prolonged nociceptive signaling (35, 36). Sympathetic-sensory crosstalk may amplify sensory neuron excitability, particularly in subchondral bone, as suggested by animal studies (10), but its temporal sequence and causal impact in human OA are not established. Sympathetic–sensory interactions are increasingly implicated as possible contributors to OA pain amplification rather than established causal drivers. Anatomical co-localization of sensory and sympathetic fibers in murine temporomandibular and knee joint tissues provides structural support for potential crosstalk (10); however, this study was performed in non-OA-affected mice and did not include pain-behavior assays. Therefore, we interpret this evidence as supporting anatomical plausibility rather than demonstrating a causal role in OA pain. In the broader context of preclinical and review-level evidence linking sympathetic activity, increased sympathetic sprouting, neurovascular remodeling, and amplified nociceptive signaling, sympathetic–sensory crosstalk may represent a suspected mechanism that enhances peripheral nociceptor responsiveness. Nevertheless, its temporal sequence, causal contribution, and relevance to human OA pain remain to be established. Sympathetic neurotransmission (e.g., norepinephrine) can enhance sensory neuron excitability and promote axonal growth, potentially preceding overt synovitis (37). This introduces an autonomic dimension to OA pain occurrence: neural regulation of vascular tone, bone microenvironment, and sensory firing can shift from homeostatic to pro-nociceptive. Together, remodeling and sympathetic coupling reduce the “distance” between pathology and pain by (i) giving nerves more access and (ii) increasing their responsiveness through autonomic modulation. These processes help explain why pain may persist despite modest inflammatory findings and why early interventions aimed at vascular-neural coupling or autonomic signaling could be particularly valuable before extensive reinnervation is established.

### 4.5 Synovial immune dysregulation and the loss of homeostatic control

The synovium is a major pain-relevant tissue in OA, but its contribution to pain is not explained simply by the presence of synovitis or by the absolute level of inflammatory markers (38). In OA, the synovium undergoes heterogeneous immune and stromal changes, including macrophage activation, altered fibroblast states, low-grade production of inflammatory mediators, and tissue-remodeling responses. These changes can influence the local joint milieu and may help determine whether structural pathology is translated into nociceptive input (39). Established clinical and imaging studies support an association between synovitis and OA pain, although the strength of this relationship varies across cohorts and disease stages (4, 19, 38). Rather than indicating a single inflammatory mechanism, these findings suggest that the synovium may act as a regulator of the local pain environment through combined effects on mediator production, tissue remodeling, and interactions with peripheral nerve endings.

At the cellular level, synovial macrophages and fibroblasts are increasingly recognized as important contributors to this process. OA synovium contains macrophage populations associated with inflammatory signaling, matrix remodeling, and crosstalk with stromal cells, while fibroblast subsets may contribute to cytokine production, extracellular matrix turnover, fibrosis-like remodeling, and maintenance of a pro-nociceptive milieu (40, 41). These observations support a model in which OA pain is influenced not only by the quantity of inflammation, but also by the qualitative state of immune-stromal regulation within the synovium. In this context, altered cellular phenotypes, persistent low-grade inflammatory signaling, and impaired resolution may be more relevant than simple elevation of conventional inflammatory markers alone.

At the same time, caution is needed in interpreting these findings. In OA, relatively well-supported observations include synovitis-pain associations (19), the presence of activated immune and stromal cell populations (39), and the ability of OA synovial fluid to enhance sensory neuron excitability in translational experimental systems (42). By contrast, broader concepts such as loss of synovial homeostatic control or defective inflammatory resolution remain emerging interpretive frameworks rather than fully established mechanisms of OA pain. Human evidence directly linking specific synovial regulatory-state alterations to pain occurrence is still limited, and much of the current literature is associative rather than causal.

Overall, current evidence supports the view that synovial immune and stromal alterations may contribute to a pain-permissive microenvironment in OA by shaping mediator release, tissue remodeling, and neuronal excitability. However, these processes should be framed as interacting and heterogeneous contributors rather than as a single, proven pathway. Further OA-specific studies integrating synovial cellular phenotyping with pain measures will be needed to clarify which immune-stromal changes are mechanistically linked to pain occurrence and persistence.

## 5 Systemic amplifiers that bias the joint toward pain

While OA pain is initiated locally within the joint, systemic factors critically shape the probability, intensity, and persistence of pain occurrence by biasing the joint microenvironment toward heightened nociceptive responsiveness. These systemic amplifiers do not necessarily generate pain independently; rather, they lower the threshold at which joint-derived signals are perceived as painful and increase the likelihood that peripheral triggers evolve into recurrent or chronic pain. Among these, metabolic dysfunction, particularly obesity, represents the most extensively studied and clinically relevant amplifier, although aging-related changes, low-grade systemic inflammation, and comorbid conditions also contribute.

### 5.1 Obesity and metabolic dysregulation as pain-amplifying states

Obesity influences OA pain through both biomechanical and non-mechanical mechanisms, with growing evidence indicating that metabolic and inflammatory pathways play a dominant role in pain occurrence. Excess adipose tissue functions as an active endocrine and immune organ, releasing adipokines, cytokines, and lipid mediators that modulate nociceptor excitability and immune responses within the joint (43). Notably, clinical studies have found that the associations between body mass index and pain severity are often stronger than those between body mass index and structural joint damage, highlighting a pain-specific effect. Adipokines such as leptin, adiponectin, and resistin can directly affect joint tissues and sensory neurons (43). Leptin, in particular, has been linked to increased pain severity and may promote nociceptive signaling by enhancing inflammatory mediator production, modulating ion channel activity, and interacting with neurotrophin pathways (44). Systemic metabolic inflammation can also prime synovial macrophages and fibroblasts toward a dysregulated state, reinforcing local immune imbalance and neurovascular remodeling (45). In this context, obesity acts less as a mechanical load and more as a systemic sensitizer, shifting joint biology toward pain permissiveness.

### 5.2 Systemic inflammation, aging, and comorbidity

Beyond obesity, low-grade systemic inflammation associated with aging (“inflammaging”), metabolic syndrome, and chronic comorbidities can further bias the joint toward pain. Circulating inflammatory mediators may not induce overt synovitis but can subtly alter immune cell function, vascular reactivity, and neuronal sensitivity within the joint. Aging-related changes in immune regulation, vascular integrity, and neural repair capacity may also reduce resilience to joint stress, making pain occurrence more likely even in response to modest local perturbations (46). Importantly, systemic amplifiers interact bidirectionally with peripheral mechanisms. By enhancing peripheral sensitization, supporting neurovascular remodeling, or impairing immune homeostasis, systemic factors increase the gain of joint-centered pain pathways. Thus, OA pain occurrence emerges from the convergence of local triggers and systemic context, highlighting the need for integrative therapeutic strategies that address both joint pathology and whole-body biological states.

Systemic factors, particularly obesity and metabolic dysfunction, appear to amplify joint pain vulnerability by biasing the local microenvironment toward heightened nociceptive responsiveness. Nonetheless, the relative contributions of metabolic, inflammatory, and aging-related systemic influences remain incompletely defined, and patient stratification in this area is still limited. These observations suggest that OA pain management may benefit from integrating joint-focused treatment with interventions targeting whole-body metabolic and inflammatory states.

## 6 Central pain persistence arising from repeated peripheral input

Pain in OA does not necessarily remain confined to the joint. While early disease is dominated by peripheral triggers of pain occurrence, repeated and sustained nociceptive input from the joint can progressively engage central pain-processing pathways, transforming episodic, state-dependent pain into persistent and difficult-to-treat pain. This transition represents a critical inflection point in OA pain biology, where mechanisms of pain generation shift from being primarily joint-centered to involving long-lasting plastic changes within the central nervous system.

Persistent peripheral input from sensitized joint nociceptors increases excitability in the spinal dorsal horn through activity-dependent synaptic plasticity (47). Enhanced glutamatergic transmission, reduced inhibitory control, and recruitment of spinal glial cells collectively lower the threshold for spinal neuron activation. As a result, nociceptive signals arising from routine joint use are amplified, prolonged, and spatially expanded (47). Clinically, this is reflected by pain that outlasts mechanical loading, occurs at rest or at night, and becomes less predictable with respect to joint activity. Over time, sustained spinal hyperexcitability can propagate to supraspinal networks involved in pain perception, affect, and modulation. Alterations in descending inhibitory and facilitatory pathways further bias the system toward pain persistence. Importantly, these central changes are not independent of the periphery: ongoing joint input is often required to maintain central sensitization, creating a bidirectional loop in which central amplification enhances the impact of residual peripheral signals (48). The transition from pain occurrence to pain persistence helps explain why structural or anti-inflammatory interventions become less effective in later-stage OA, and why some patients develop widespread pain or nociplastic features. It also highlights a window of opportunity in which targeting peripheral drivers early may prevent or attenuate maladaptive central plasticity. Understanding this peripheral-to-central continuum is therefore essential for developing stage-specific, mechanism-based strategies to treat and prevent chronic OA pain.

Repeated peripheral nociceptive input is increasingly recognized as a driver of central pain persistence in OA, helping explain the transition from intermittent pain to more refractory and widespread pain states. However, the thresholds, timing, and reversibility of these central changes remain unclear, especially in relation to specific peripheral phenotypes. Clinically, this underscores the importance of identifying and treating peripheral pain drivers early, before central amplification becomes self-sustaining.

## 7 Therapeutic implications targeting pain occurrence mechanisms

Understanding OA pain as a biologically regulated process of pain occurrence, rather than a passive consequence of structural damage, has important therapeutic implications. However, the translational maturity of candidate interventions differs substantially across mechanisms. Some approaches, such as NGF pathway inhibition, have been clinically tested in OA and provide direct support for mechanism-based analgesia, whereas others are supported mainly by preclinical or translational studies, and still others remain emerging conceptual strategies with limited OA-specific validation. Accordingly, the therapeutic implications discussed below should be interpreted in an evidence-calibrated manner: they represent a spectrum ranging from clinically examined approaches to promising but still exploratory mechanistic targets. This distinction is important to maintain consistency with the current limitations of the field, including phenotype heterogeneity, incomplete causal inference, and the persistent translational gap between experimental models and human OA pain.

### 7.1 Targeting chemical triggers and joint acidosis

One of the most direct ways to modulate pain occurrence is to interfere with chemical signals that activate nociceptors. Proton-sensing pathways represent an attractive target, as joint acidosis can both trigger and amplify pain (4). Inhibition of acid-sensing ion channels (e.g., ASIC3), modulation of TRP channels such as TRPV1 or TRPA1, and targeting proton-sensing G protein-coupled receptors may reduce nociceptor depolarization in acidic microdomains (49, 50). Beyond pH itself, blocking key algogenic mediators-such as prostaglandins, cytokines, or lipid mediators-can dampen the chemical “gain” of the joint microenvironment (51). Importantly, these approaches aim not merely to suppress inflammation but to normalize nociceptor excitability, potentially reducing episodic pain flares linked to metabolic or mechanical stress.

### 7.2 Modulating peripheral neuronal sensitization

Peripheral sensitization is a major driver of more frequent pain occurrence and remains an attractive therapeutic entry point, but the supporting evidence varies across targets. Among the strongest proof-of-principle examples, NGF pathway inhibition has shown that reducing peripheral nociceptor sensitization can produce clinically meaningful analgesia in OA, although safety concerns have limited broad clinical adoption and underscore the need for more selective or context-dependent approaches. Other strategies, including modulation of voltage-gated sodium channels such as Nav1.7 and Nav1.8, are mechanistically compelling because they directly regulate nociceptor excitability, but OA-specific clinical validation remains limited (52). In this context, the recent clinical success of suzetrigine, a peripherally acting sodium channel-targeting analgesic, is relevant as proof-of-principle that peripherally restricted sodium channel modulation can yield meaningful pain relief in humans, even though it has not yet been studied in OA (53). Additional approaches targeting calcium channels or intracellular signaling downstream of neurotrophin and cytokine receptors are currently supported mainly by preclinical and translational data. Overall, therapies aimed at peripheral sensitization should be viewed as a heterogeneous group spanning clinically tested, translationally supported, and still-emerging strategies, rather than as a uniformly validated therapeutic class.

### 7.3 Interfering with neurovascular remodeling and autonomic amplification

Structural and autonomic contributors to pain occurrence offer additional therapeutic targets. Interventions that limit pathological angiogenesis or nerve ingrowth may reduce the expansion of nociceptive networks within the joint. Modulating vascular-neural coupling could help limit the establishment of persistent peripheral nociceptive input (6, 35). Similarly, targeting sympathetic-sensory interactions, for example through β-adrenergic modulation or regulation of catecholamine signaling, may attenuate early neural amplification, particularly in subchondral bone (54). These approaches emphasize timing, as preventing or reversing early remodeling is likely to be more effective than attempting to eliminate established reinnervation.

### 7.4 Restoring synovial immune homeostasis

Restoring synovial immune homeostasis plays a crucial role in the treatment of OA, especially in alleviating pain associated with immune dysfunction. Immune dysregulation in the synovium is a key factor in the progression of OA, manifesting as impaired immune cell function and persistent inflammation. Studies have shown that immune cells, such as macrophages, fibroblasts, and T cells, become dysfunctional in OA, leading to chronic low-grade inflammation and inadequate tissue repair, which in turn exacerbates joint pain (55). Therefore, restoring immune homeostasis within the synovium, particularly by modulating the function of these immune cells, emerges as a promising therapeutic approach. Current strategies involve promoting the polarization of macrophages toward the anti-inflammatory M2 phenotype, thereby reducing inflammation and enhancing tissue repair (56); adjusting fibroblast function to reduce fibrosis and inflammation while improving joint structural repair (57); and enhancing the activity of regulatory T cells (Tregs) to restore immune tolerance and suppress excessive immune responses (58). Additionally, targeting key cytokines, such as TNF-α and IL-1β, is being explored as a way to reduce synovial inflammation and pain. Despite the potential of immune-based therapies, several challenges remain, such as OA’s heterogeneity and patient variability, the need for precise treatment, and controlling potential side effects. Future research must delve deeper into the changes in synovial immune function and their impact on OA pain, focusing on how to restore immune homeostasis without suppressing the immune system’s normal functions, ultimately enabling more effective pain management and disease control.

### 7.5 Integrating peripheral and central strategies

Finally, targeting the mechanisms that trigger pain occurrence should be seen as a complementary approach to, rather than a replacement for, therapies that address central pain processing (59). Focusing on reducing peripheral nociceptive input in the early stages of OA can prevent the escalation of pain and may slow the transition to central sensitization, where the central nervous system becomes increasingly involved in pain perception. By managing the frequency and intensity of pain signals originating in the joint, early-stage interventions can help minimize the need for more aggressive centrally acting analgesics, thus preserving their effectiveness for the later stages of the disease. This integrated approach, which targets both peripheral and central mechanisms of pain, emphasizes the importance of intervening early in the disease progression to prevent chronic pain and its associated long-term effects. Moreover, this strategy supports a stage-specific, mechanism-based approach to OA pain management, where the choice of therapy is guided by an understanding of the dominant pain drivers at each stage of the disease. By matching the right treatment to the underlying pain mechanism, this approach enhances the likelihood of more effective and sustainable pain relief, ultimately improving patient outcomes over time.

Overall, current evidence supports targeting peripheral mechanisms of pain occurrence as a promising strategy for improving OA pain management, particularly in earlier disease stages before persistent central amplification becomes established. However, the clinical effectiveness, safety, and optimal timing of many mechanism-based interventions remain uncertain, and their benefits may differ across patient phenotypes and disease stages. Clinically, these considerations support a stage-specific and mechanism-informed approach, in which therapies are matched as closely as possible to the dominant drivers of pain.

## 8 Cross-cutting limitations and unresolved questions

Across mechanistic domains, several overarching limitations temper current interpretations of OA pain occurrence. First, much of the available evidence is cross-sectional (4, 5, 19, 20), limiting causal inference and making it difficult to determine whether identified biological changes precede increases in pain frequency or represent adaptations to sustained nociceptive input. For example, emerging biomarkers such as IL-6 and TNF-α are being investigated in clinical trials to assess their role in predicting pain intensity and inflammation in OA (1, 2, 6). These biomarkers could potentially help in stratifying patients and identifying those who are more likely to benefit from specific treatments. However, challenges remain in standardizing their use and validating their effectiveness across different patient populations. Second, pain intensity remains the dominant clinical outcome in both observational studies and trials (2, 3, 18, 19), whereas measures more closely aligned with pain occurrence-such as flare frequency, temporal variability, rest or nocturnal pain, and recovery time after activity-are less consistently captured. This mismatch may obscure mechanisms that primarily influence the probability and timing of pain episodes rather than overall severity. Third, substantial heterogeneity exists across OA stages, joint sites, comorbid conditions, and prior treatments, yet many studies do not stratify by phenotype, potentially contributing to inconsistent findings (6, 11, 16, 35). Fourth, translational gaps persist between experimental models and human disease, particularly regarding neurovascular remodeling, immune dysregulation, and metabolic influences, which may operate differently across species and disease timelines (36, 60, 61). Finally, peripheral and central processes are frequently inferred indirectly and often co-occur, complicating efforts to disentangle their relative contributions to pain trajectories (14, 62, 63). Addressing these cross-cutting challenges will require longitudinal, stage-stratified human cohorts; integration of imaging, molecular, and sensory phenotyping; and outcome measures that move beyond intensity to capture the dynamics of pain occurrence and persistence.

## 9 Future directions

Future work in OA pain should prioritize clarifying which mechanisms are causal drivers of pain occurrence versus secondary consequences of ongoing nociception, particularly for neurovascular remodeling, sympathetic-sensory interactions, and synovial immune dysregulation (6, 10, 36, 39). Longitudinal human cohorts and time-resolved experimental models are needed to define the temporal sequence of peripheral triggers and to identify therapeutic windows before central amplification becomes dominant. Another major direction is developing mechanism-based patient stratification, integrating clinical pain patterns, quantitative sensory testing, imaging markers (e.g., synovitis, bone marrow lesions, vascular features), and molecular signatures (including acidosis- and neurotrophin-related pathways) to match therapies to dominant drivers. Key open questions also include how specific patterns of peripheral input shape central plasticity, whether late-stage peripheral interventions can reverse established central sensitization, and which biomarkers can reliably capture pain “occurrence” and predict transition to persistence. Finally, translational progress will require outcome measures that move beyond pain intensity to quantify pain frequency, fluctuation, and persistence, enabling trials to test whether targeting joint-centered mechanisms can prevent chronic, refractory OA pain.

## 10 Conclusion

OA pain arises from active joint–nerve mechanisms rather than cartilage loss alone. In innervated tissues, algogenic mediators and acidosis trigger nociceptors, peripheral sensitization increases pain frequency, and neurovascular, autonomic, and synovial immune changes sustain a pain-permissive joint state. Persistent peripheral input can then drive central amplification and chronicity. Mechanism- and stage-matched therapies that target these early drivers may improve durable pain control.
